# Evaluating post-vaccine expansion patterns of pneumococcal serotypes

**DOI:** 10.1016/j.vaccine.2020.10.045

**Published:** 2020-11-17

**Authors:** Maile T. Phillips, Joshua L. Warren, Noga Givon-Lavi, Adrienn Tothpal, Gili Regev-Yochay, Ron Dagan, Daniel M. Weinberger

**Affiliations:** a Department of Epidemiology of Microbial Diseases, Yale School of Public Health, New Haven, CT, United States; b Department of Biostatistics, Yale School of Public Health, New Haven, CT, United States; c Faculty of Health Sciences, Ben-Gurion University of the Negev, Be'er Sheva, Israel; d Semmelweis University, Institute of Medical Microbiology, Budapest, Hungary; e Sackler Faculty of Medicine, Tel Aviv University, Tel Aviv, Israel; f Infection Prevention & Control Unit, Sheba Medical Center, Tel-Hashomer, Israel

## Abstract

**Background:**

*Streptococcus pneumoniae* remains a leading cause of morbidity and mortality. Pneumococcal conjugate vaccines (PCVs) are effective but target only a fraction of the more than 90 pneumococcal serotypes. As a result, the introduction of PCVs has been followed by the emergence of non-vaccine serotypes. With higher-valency PCVs currently under development, there is a need to understand and predict patterns of serotype replacement to anticipate future changes.

**Methods:**

In this study, we fit a hierarchical Bayesian regression model to evaluate patterns of change in serotype prevalence post-PCV introduction in Israel from 2009 to 2016.

**Results:**

We found that the assumption that non-vaccine serotypes increase by the same proportion overestimates changes in serotype prevalence in Jewish and Bedouin children. Furthermore, pre-vaccine prevalence was positively associated with increases in prevalence over the study period. From our analyses, serotypes 12F, 8, 16F, 33F, 9N, 7B, 10A, 22F, 24F, and 17F were estimated to have gained the most cases of invasive pneumococcal disease through serotype replacement in the Jewish population. However, this model also failed to quantify some additional cases gained, suggesting that changes in carriage in children alone may be insufficient to explain serotype replacement in disease.

**Conclusions:**

Understanding of serotype replacement is important as higher-valency vaccines are introduced.

## Highlights

• The assumption that serotypes increase by the same amount is an overestimation.
• Pre-vaccine prevalence is positively associated with increases in prevalence.
• Changes in carriage in children alone does not explain disease serotype replacement.
• This model can quantify changes in serotype replacement in other populations.
• This model may help to optimize future serotype compositions.

## 1 Introduction

*Streptococcus pneumoniae* (pneumococcus) is a leading cause of morbidity and mortality globally and causes a range of diseases [1]. Nasopharyngeal carriage of pneumococcus is a precursor for disease [2]. Carriage is common in children, who are the main reservoir of transmission [2], [3]. The likelihood of developing disease following exposure depends on bacterial (serotype and virulence factors) and host factors (age, immunocompetence, and recent viral infection) [4], [5].

Pneumococcal conjugate vaccines (PCVs) protect against carriage and infections due to serotypes targeted by the vaccine [6], [7], [8], [9]. However, PCVs target only a fraction of the 90+ serotypes. As a result, despite an overall decline in disease, the PCV introduction is followed by non-vaccine serotype (NVT) emergence [6], [10], [11]. This ‘serotype replacement’ has reduced the overall impact of PCVs on disease rates and prompted the development of PCVs that target larger numbers of serotypes. Several new PCVs are under development that target 15, 20, or more serotypes [12], [13], [14]. These vaccines will again likely disrupt the balance of serotypes in the nasopharynx and lead to further serotype replacement. As these next-generation conjugate vaccines move towards licensure, it is important to understand likely patterns of serotype replacement so that the marginal benefit of higher-valency vaccines can be anticipated. This is useful as countries decide which product to recommend in a given setting.

When evaluating the potential impact of new vaccines, there is a need to predict overall benefits, which are influenced by both declines in disease incidence due to vaccine-targeted serotypes (VTs) and by increases in disease incidence due to NVTs. At the same time, it is important to predict which serotypes are likely to emerge to help with future adjustments to the vaccine. To produce such projections, it is necessary to make assumptions about which serotypes are likely to increase and by how much. There are several proposed frameworks for accomplishing this goal. Nurhonen and Auranen predicted the impact of alternative PCV formulations with the assumption that all NVTs increase by the same proportion following vaccine introduction and that the invasiveness of NVTs as a group is constant over time [15]. This approach can successfully capture the overall benefit by lumping VTs and NVTs into groups [16], [17], [18]. However, when the goal is to examine emerging serotypes, it is important to consider individual serotypes. Recent work has used genomic data with models of negative frequency-dependent selection to quantify post-vaccine expansion patterns [19], [20]. However, whole genome sequence data are not always widely available, and their interpretation is complex. A complementary approach would be to identify characteristics of serotypes that emerge following vaccine introduction and to use this information to inform projections.

In this study, we characterized NVT expansion patterns among carriers following the PCV introduction in Israel. We evaluated correlates of serotype expansion, compared the expansion patterns to those that would be expected if the assumption that all NVTs expand by the same amount were correct, and assessed the implications for disease projections if this assumption were wrong. Finally, we provided a framework to rank serotypes not currently included in PCV13 based on the likelihood that they may be important causes of disease in the future.

## 2 Material and methods

### 2.1 Data, setting, & participants

PCV7 was introduced for use in Israel in the national immunization program in July 2009 with a catch-up program in children <2 years old and was replaced by PCV13 in November 2010 without further catch-up. PCV13 uptake increased to approximately 80% by June 2011, 90% by December 2012, and 95% by June 2014 for children 7–11 months of age receiving ≥2 PCV doses [21], [22], [23]. Since its incorporation into the country’s immunization schedule, PCV uptake has been similarly high among Jewish and Bedouin children, with over 90% rates of children under 2 years of age having had ≥3 doses by 2014 [21].

Carriage data were collected as part of surveillance in Israel from November 2009-June 2016, described in detail elsewhere [23]. Every weekday during the study period, nasopharyngeal cultures were collected from the first four Jewish and four Bedouin children under five presenting at a pediatric emergency department in Be’er Sheva, Israel for any reason. Ethnicity, pneumococcal culture result, pneumococcal serotype (if applicable), clinical diagnosis, number of PCV doses received to date (PCV7 and/or PCV13), and the swab year and month were recorded.

Invasive pneumococcal disease (IPD) data for individuals of all ages were also collected during the same time period as part of nationwide surveillance. Variables were collected regarding age group (<5, 5–17, 18–39, 40–64, and 65+ years), serotype, date, and number of swabs. Both the carriage and IPD datasets were aggregated into counts by serotype and epidemiological year (seven July-June years); the number of swabs negative for pneumococcus in the carriage dataset was also tallied. Year 0 in the carriage dataset is only ¾ of an epidemiological year, due to the fact that data collection started in November that year.

### 2.2 Other data

Additional serotype-specific characteristics were used to evaluate associations with changes in prevalence. These variables included relative density of growth [24], case fatality rate, negative charge carbon, total carbon per polysaccharide repeat, and capsule thickness [25]. In instances of missing data, missing values were imputed assuming the data were missing completely at random (S1 Text).

### 2.3 Carriage model

With the goal of quantifying changes in carriage serotype prevalence and evaluating correlates changes in prevalence, we fit a hierarchical Bayesian regression model to the data. The data for individual serotypes were sparse, so we used a hierarchical prior structure to provide stabilized estimates [26]. In this model, pneumococcal carriage at time (epidemiological year) *t* follows a multinomial distribution with the number of successes being the number of detections of each NVT, and the number of trials being the total number of nasopharyngeal swabs at time *t*. The “reference” category in this model includes all negative swabs and VTs. The change in prevalence in the post-vaccine period varies by serotype. In this model, time is a three-category variable, corresponding to the pre- (November 2009-June 2010), early post-PCV (July 2010-June 2012), and late post-PCV (July 2012-June 2016) vaccination periods. This time structure provides a better fit compared to other functions of time, and aligns with the epidemiological years of introduction and change in the vaccination periods. The three-category structure of time allows serotype prevalence to level out to its final post-vaccine prevalence, facilitating a better comparison of pre- to post-vaccine prevalence. The model is defined as:

$${n.carr}_{t}\;Multinomial\left({N.swab}_{t};{\mathrm{pre}v_{\mathrm{rt}},prev}_{1t},{\mathrm{prev}}_{2t},\cdots ,{\mathrm{prev}}_{\mathrm{mt}}\right)$$

$$\ln\left(\frac{{\mathrm{prev}}_{\mathrm{it}}}{{\mathrm{prev}}_{\mathrm{rt}}}\right)=\beta_{0,i}+\beta_{1,i}*time_{1,t}+\beta_{2,i}*time_{2,t}$$

$$\beta_{2,i}=\mu_{0,i}+\sum \alpha_{j}x_{\mathrm{ij}}$$

*n.carr_t_* = pneumococcal carriage count at time period *t*
*N.swab_t_* = total # swabs at time period *t*
*prev_it_* = pneumococcal prevalence for NVT *i* at time
period *t* (m = 54 NVTs)
$\mathrm{pre}v_{\mathrm{rt}}$ = pneumococcal prevalence from the “reference” group at time
period *t*
*i* = 1, 2, …, m
*t* = 0, 1, …, 6 (year)
*time_1_* = 0, 1, 1, 1, 1, 1, 1
*time_2_* = 0, 0, 0, 1, 1, 1, 1
x*_ij_* = the *j*^th^ serotype-level covariate for NVT *i*

All prior distributions were weakly informative. The intercepts (relative prevalence at *time 0*) and slopes (early and late post-vaccine prevalence compared to *time 0*) were allowed to vary by serotype and were estimated hierarchically by having the serotype-specific parameters centered around global parameters that were common to all NVTs. Conjugate priors were used in all cases except for the standard deviation parameters, where uniform priors were used [27].

Different assumptions about model structure and covariates were evaluated for this model. Variations in random and fixed effects were considered, different structures of time were evaluated, and covariance matrices were applied to model independence and dependence of the intercepts and slopes. The best model formulation was chosen using the optimal deviance information criterion (DIC) [28], using decreases of at least 10 to be considered an improved model. With Markov chain Monte Carlo sampling techniques, we obtained 150,000 posterior samples from the joint posterior distribution following a burn-in period of 10,000 iterations. The Gelman-Rubin [29] and Geweke [30] diagnostics were used to investigate convergence of individual parameters and effective sample sizes were calculated to ensure we collected enough posterior samples post-convergence for accurate inference. Additional details about the model, including structure and model diagnostics, can be found in the supplementary text (S1 Text).

Serotype-specific prevalence, relative risk ratios (RRRs; the multiplicative change for a one-year increase for a specific serotype prevalence relative to the reference prevalence), slopes, and intercepts were estimated from the final model. Where relevant, these estimates were compared to the corresponding values from the constant proportional change model.

### 2.4 Linking carriage with disease

We sought to quantify the impact of serotype replacement on IPD. This is challenging because many serotypes have secular trends or exhibit epidemic patterns even in the absence of vaccines. Simply comparing the cases of IPD before and after vaccination can sometimes be misleading. Instead, we chose a more stable approach which used the estimates of the serotype-specific prevalence ratios from our carriage model to estimate the additional cases gained as a result of increased carriage in children (*cases.gained.explained*). The number of cases that would be expected in the absence of changes in carriage in children is calculated by dividing the observed number of cases at a given time point by the prevalence ratio for the same serotype. The cases gained (explained by increased carriage) estimate is calculated by subtracting this counterfactual for the number of cases from the observed number of cases for each serotype, age category and time:

$$\mathrm{cases}.gaine{d.explained}_{\mathrm{serotype},age,time}=observed.cases_{\mathrm{serotype},age,time}-\frac{{\mathrm{observed}.cases}_{\mathrm{serotype},age,time}}{{\mathrm{prev}.ratio}_{\mathrm{serotype},time}}$$

where the prevalence ratios (*prev.ratio*) were estimated from the model. We also calculated the number of cases gained that were not explained by increased carriage in children (e.g., could be due to secular trends; *cases.gained.unexplained*), by subtracting the observed number of IPD cases at *time 0* from the number of cases that would have been expected in the absence of changes in carriage in children (i.e., the number of IPD cases in the first time period):

$$\mathrm{case}{s.gained.unexplained}_{\mathrm{serotype},age,time}=\frac{{\mathrm{observed}.cases}_{\mathrm{serotype},age,time}}{{\mathrm{prev}.ratio}_{\mathrm{serotype},time}}-observed.cases_{\mathrm{serotype},age,time0}$$

Using the values of cases gained as a result of increased carriage in children, we sorted the serotypes from highest to lowest. All of these calculations were carried out using results from the Jewish carriage model only, since the IPD dataset predominantly included Jewish individuals.

In some instances, the expected number of IPD cases exceeded the number of observed cases. In such cases, we adjusted the expected number of cases to be the number of observed cases. In other words, we let the maximum number of expected cases be the number of observed cases, and zero cases gained in that instance.

To assess whether changes in carriage in younger children or older children better correlated with changes in IPD in adults, we additionally fit the carriage model separately for younger children (<12 months) and older children (different subsets of 24–60-month-old children), and compared the number of unexplained versus explained cases gained between the two age groups.

All analyses were performed using R version 3.4.0 [31]. The carriage model was fit using JAGS version 4.3.0 [32]. All code, with simulated data, can be found online at https://github.com/mailephillips/post-pcv-expansion.

## 3 Results

### 3.1 Characteristics of the data

The Israel carriage dataset contained 10,396 observations, with 57.1% of samples from Bedouin children and the rest from Jewish children. Of all swabs, 47.7% were positive for pneumococcus, with 42.6% positive among Jewish children and 51.5% positive among Bedouin children. In 2009, just after vaccine introduction, 63.5% of colonized children had a VT and 36.5% had a NVT. The most commonly-carried non-PCV13 serotypes in 2009 were 15B/C, 15A, 16F, 10A, 38, 35B, 23B, 23A, 21, and 10B.

### 3.2 Serotype expansion patterns in pneumococcal carriage

**Fig. 1:**
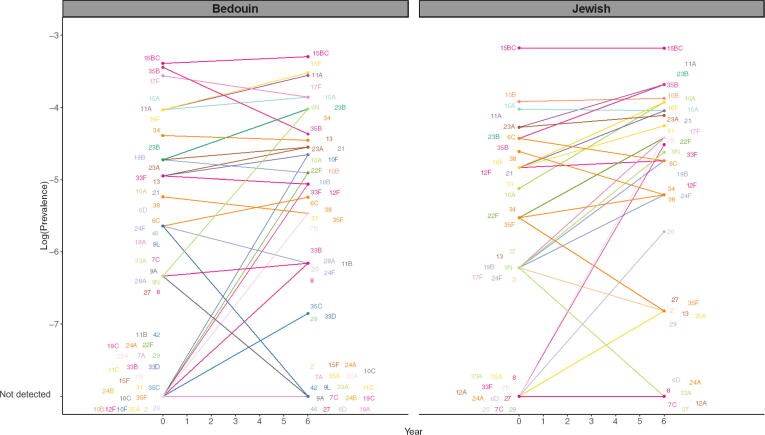
Pre-vaccine vs. post-vaccine pneumococcal prevalence of non-vaccine types for children under 5 in Israel, November 2009-July 2016. The observed log-prevalence of pneumococcus for each serotype is shown at Year 0 and Year 6, separately for Jewish and Bedouin children. If a serotype increased from or decreased to 0, the log prevalence is shown as “not detected” at the bottom of the graph.

**Fig. 2:**
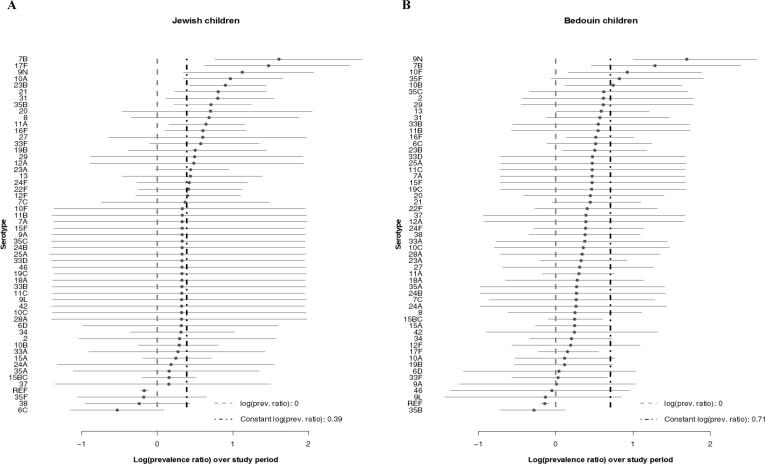
Log(prevalence ratios) from the carriage models for children under 5 in Israel, November 2009-June 2016. Log-transformed ratios comparing late post- to pre-vaccine prevalence (last year/first year) are shown for each serotype with 95% credible intervals from the Jewish (A) and Bedouin (B) carriage models. The serotypes are shown from highest to lowest log prevalence ratios.

Some typical assumptions when projecting serotype replacement are that all NVTs increase by the same amount (i.e., same prevalence ratio), that the rank order of NVTs is maintained following vaccine introduction, and that NVTs completely replace VTs in carriage. When we compare the observed pre- to post-vaccine prevalence, serotypes increase by different proportions and do not maintain rank order, suggesting that the assumption of constant prevalence ratio does not hold (Fig. 1). Our analysis suggests that instead some serotypes expand more than expected, and others remain stable or even decline (Fig. 2).

The serotypes with the largest relative increase in carriage prevalence among Jewish children, from highest to lowest, were 7B, 17F, 9N, 10A, 23B, 21, 31, 35B, 20, and 8. Among Bedouin children, the largest relative increases were seen in 9N, 7B, 10F, 35F, 10B, 35C, 2, 29, 13, and 31 (Fig. 2, Table S2). The majority of the prevalence ratios were larger than one in both models (Jewish median prevalence ratio: 1.35, Bedouin median: 1.36). In both models, many serotypes had changes that were smaller than expected based on the model assuming proportional increases (constant proportional increase: 1.48 Jewish, 2.03 Bedouin). Serotype-specific intercepts and other serotype-specific parameters estimated from the model can be found in Table S2 and Figures S5-7.

### 3.3 Correlates of serotype-specific changes

While serotype-specific prevalence of the non-PCV13 serotypes mostly increased after vaccination, the degree to which each serotype changed varied. The distribution of the change in prevalence post-vaccination was skewed, but we were able to predict where on the distribution each serotype was located using serotype-specific characteristics. The best correlate of the post-vaccine increase was pre-vaccine prevalence. The relationship between pre-vaccine prevalence and the change in serotype prevalence over time improved model fit when included with a covariance matrix compared to all other combinations of predictors. When fit to carriage data from Jewish children, the model with the covariance matrix was the best model, based on DIC. When fit to carriage data from Bedouin children, the model with independent slopes (no predictors) and the model with the covariance matrix both fit the data best based on DIC. There was a positive association between the pre-vaccine and the change to late post-PCV prevalence in the Jewish dataset (0.04, CI(CI): −0.68, 0.68), and a negative association in the Bedouin dataset (-0.21, CI: −0.69, 0.34). Combinations of relative density, case fatality rate, negative charge carbon, total carbon per polysaccharide repeat, and capsule thickness were not as strongly associated with the prevalence ratio and resulted in worse model fits in both dataset.

### 3.4 Ranking serotypes based on estimated cases gained

**Fig. 3:**
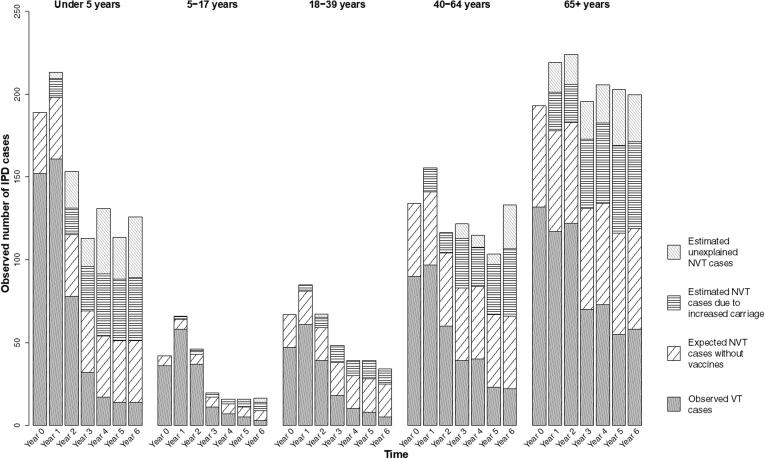
Estimated additional cases gained as a result of increased carriage in children under 5 in Israel, July 2009-July 2016. Results are shown for each age group and year of the study. Each full bar represents the total number of observed IPD cases for that stratum. The different patterned portions of the bars denote the fraction of total cases attributable to different groups of serotypes: observed vaccine-targeted (VT) serotype cases (solid gray), expected non-vaccine-targeted (NVT) serotype cases had PCV13 not been introduced (diagonal black lines), estimated additional cases gained as a result of increased carriage in children, explained by this model (horizontal black lines), and estimated additional cases gained that are not explained by this model (gray diagonal gray lines).

From 2009 to 2016, there were 3968 cases of IPD. We estimate that during this period, there was an excess of 624 (CI: 397, 850) cases gained across all age groups as a result of increased carriage of NVTs in children (Fig. 3, Table 1). From highest to lowest, serotypes 12F, 8, 16F, 33F, 9N, 7B, 10A, 22F, 24F, and 17F were estimated to have gained the most cases of IPD through serotype replacement (each with an estimated 21 to 112 additional cases gained). These 10 serotypes combined had a total of 1223 reported IPD cases during this period. An estimated 446 of these cases could be attributed to increases in carriage prevalence (Table 1).

**Table 1:** Model Estimates and Cases Gained Through Serotype Replacement over 6 Years among Jewish Individuals in Israel (July 2009-June 2016), Ranked from Most to Least Cases Gained in All Age Categories. The results of the observed carriage at time 0, observed prevalence ratio (time_6_/time_0_), model prevalence ratio (time_6_/time_0_), and estimated cases gained through serotype replacement by age groups are shown for each of the serotypes used in the carriage model. Serotypes are ordered from most to least cases gained among all ages. Note that the total cases gained in the last row adds up to more than the number of cases above for each serotype, because the serotypes shown are only those used in the carriage model. The values for total cases gained also included calculations for additional serotypes (not shown) that were responsible for IPD, but were not present in carriage in children under 5 during this time period.

	***Observed***		***Model***		***Cases gained over study period, by age in years***											
**Serotype**	**<5 carr. (t_0)_**	**PR (t_6_/t_0_)**	**PR (t_6_/t_0_)**		**<5**		**5**–**17**		**18**–**39**		**40**–**64**		**65+**		**All Ages**	
			**Med.**	**CrI**	**Med.**	**CrI**	**Med.**	**CrI**	**Med.**	**CrI**	**Med.**	**CrI**	**Med.**	**CrI**	**Med.**	**CrI**
**12F**	4	1.10	1.48	0.75, 2.98	58	0, 114	3	0, 6	8	0, 15	20	0, 39	22	0, 44	112	0, 219
**8**	0	NA**	1.96	0.69, 6.44	4	0, 7	3	0, 5	14	0, 23	29	0, 49	23	0, 38	72	2, 122
**16F**	4	2.48	1.82	1.10, 3.29	6	1, 9	0	0, 0	3	1, 4	14	4, 24	27	7, 45	50	13, 82
**33F**	0	NA*	1.78	0.90, 3.95	20	1, 35	0	0, 1	3	0, 5	5	1, 9	11	1, 19	39	4, 69
**9N**	1	1.00	3.08	1.39, 8.09	5	2, 7	1	1, 2	4	2, 5	9	4, 12	15	6, 20	35	15, 46
**7B**	0	NA*	4.98	2.13, 15.25	10	6, 11	0	0, 0	1	1, 1	6	4, 7	15	10, 18	32	21, 38
**10A**	3	3.30	2.62	1.40, 5.28	10	4, 14	0	0, 0	2	1, 2	8	4, 11	11	5, 15	31	14, 42
**22F**	2	3.03	1.50	0.79, 3.10	5	0, 11	1	0, 2	2	0, 3	9	0, 17	11	0, 22	28	1, 55
**24F**	1	2.75	1.52	0.75, 3.32	7	0, 15	1	0, 2	2	0, 4	6	0, 12	12	0, 24	28	1, 56
**17F**	1	6.05	4.32	1.84, 13.00	3	2, 4	0	0, 1	2	1, 3	5	3, 6	10	6, 13	21	12, 27
**23B**	6	2.11	2.46	1.52, 4.19	4	2, 5	2	1, 2	0	0, 1	4	2, 5	12	7, 15	21	12, 28
**15A**	9	0.98	1.28	0.82, 2.06	3	0, 8	0	0, 1	0	0, 0	4	0, 9	13	0, 28	21	0, 46
**31**	4	1.79	2.21	1.10, 4.62	1	0, 1	1	0, 1	1	0, 2	6	1, 8	12	3, 19	21	4, 31
**11A**	7	1.81	1.90	1.17, 3.22	2	0, 3	1	0, 1	2	1, 4	7	2, 10	8	2, 12	19	6, 29
**35B**	6	2.11	2.03	1.23, 3.50	2	1, 3	0	0, 0	1	0, 1	3	1, 5	11	4, 16	17	6, 25
**15BC**	21	1.00	1.16	0.81, 1.66	8	0, 20	0	0, 0	1	0, 2	3	0, 7	4	0, 10	16	0, 39
**10B**	10	1.05	1.33	0.76, 2.27	5	0, 10	0	0, 1	0	0, 1	1	0, 2	6	0, 13	12	0, 27
**2**	1	0.55	1.32	0.35, 4.71	3	0, 8	2	0, 6	2	0, 6	3	0, 11	1	0, 3	11	0, 34
**23A**	7	1.18	1.55	0.97, 2.61	1	0, 2	0	0, 0	0	0, 1	4	0, 7	5	0, 8	10	1, 17
**34**	2	1.38	1.36	0.69, 2.80	1	0, 1	0	0, 1	0	0, 1	1	0, 2	6	0, 13	7	0, 17
**27**	0	NA*	1.83	0.52, 7.33	6	0, 12	0	0, 1	0	0, 0	0	0, 0	0	0, 0	6	0, 12
**38**	5	0.55	0.78	0.38, 1.56	1	0, 4	0	0, 0	0	0, 0	1	0, 4	3	0, 10	5	0, 17
**13**	1	0.55	1.54	0.62, 4.00	0	0, 1	0	0, 1	0	0, 0	2	0, 4	2	0, 5	5	0, 10
**21**	4	2.20	2.22	1.25, 4.20	3	1, 5	0	0, 0	0	0, 0	0	0, 0	0	0, 0	3	1, 5
**6C**	6	0.73	0.58	0.32, 1.09	1	0, 2	0	0, 0	0	0, 1	0	0, 2	2	0, 5	3	0, 9
**20**	0	NA*	1.99	0.62, 7.66	0	0, 0	0	0, 0	1	0, 1	1	0, 1	2	0, 3	3	0, 5
**29**	0	NA*	1.60	0.40, 6.86	1	0, 3	0	0, 0	0	0, 0	0	0, 0	0	0, 1	1	0, 3
**35F**	2	0.28	0.83	0.34, 1.90	0	0, 1	0	0, 0	0	0, 1	0	0, 1	1	0, 5	1	0, 8
**9A**	0	NA**	1.35	0.24, 6.93	0	0, 1	0	0, 0	0	0, 1	0	0, 1	0	0, 1	1	0, 3
**24B**	0	NA**	1.34	0.23, 6.87	1	0, 2	0	0, 0	0	0, 0	0	0, 1	0	0, 0	1	0, 3
**18A**	0	NA**	1.35	0.23, 6.77	0	0, 0	0	0, 0	0	0, 0	1	0, 2	0	0, 1	1	0, 3
**25A**	0	NA**	1.35	0.24, 6.75	0	0, 0	0	0, 0	0	0, 0	0	0, 1	0	0, 1	1	0, 2
**10F**	0	NA**	1.35	0.24, 6.91	0	0, 0	0	0, 0	0	0, 1	0	0, 0	0	0, 1	1	0, 2
**33A**	0	NA**	1.29	0.40, 4.06	0	0, 1	0	0, 0	0	0, 0	0	0, 0	0	0, 0	0	0, 1
**19B**	1	4.40	1.63	0.67, 4.23	0	0, 0	0	0, 0	0	0, 1	0	0, 0	0	0, 0	0	0, 1
**6D**	0	NA**	1.36	0.36, 4.87	0	0, 0	0	0, 0	0	0, 0	0	0, 0	0	0, 1	0	0, 1
**9L**	0	NA**	1.35	0.23, 7.02	0	0, 0	0	0, 0	0	0, 0	0	0, 1	0	0, 0	0	0, 1
**37**	1	0.00	1.13	0.25, 4.35	0	0, 0	0	0, 0	0	0, 1	0	0, 1	0	0, 0	0	0, 2
**28A**	0	NA**	1.35	0.24, 6.83	0	0, 0	0	0, 0	0	0, 0	0	0, 0	0	0, 1	0	0, 1
**11C**	0	NA**	1.34	0.23, 6.88	0	0, 0	0	0, 0	0	0, 0	0	0, 0	0	0, 1	0	0, 1
**35A**	0	NA*	1.16	0.32, 3.77	0	0, 0	0	0, 0	0	0, 0	0	0, 0	0	0, 1	0	0, 1
**10C**	0	NA**	1.34	0.24, 6.90	0	0, 0	0	0, 0	0	0, 0	0	0, 0	0	0, 0	0	0, 0
**11B**	0	NA**	1.34	0.24, 6.85	0	0, 0	0	0, 0	0	0, 0	0	0, 0	0	0, 0	0	0, 0
**12A**	0	NA**	1.60	0.40, 6.97	0	0, 0	0	0, 0	0	0, 0	0	0, 0	0	0, 0	0	0, 0
**15F**	0	NA**	1.35	0.24, 7.00	0	0, 0	0	0, 0	0	0, 0	0	0, 0	0	0, 0	0	0, 0
**19C**	0	NA**	1.34	0.24, 6.88	0	0, 0	0	0, 0	0	0, 0	0	0, 0	0	0, 0	0	0, 0
**24A**	0	NA**	1.17	0.26, 4.59	0	0, 0	0	0, 0	0	0, 0	0	0, 0	0	0, 0	0	0, 0
**33B**	0	NA**	1.35	0.24, 6.87	0	0, 0	0	0, 0	0	0, 0	0	0, 0	0	0, 0	0	0, 0
**33D**	0	NA**	1.35	0.24, 6.84	0	0, 0	0	0, 0	0	0, 0	0	0, 0	0	0, 0	0	0, 0
**35C**	0	NA**	1.34	0.24, 6.89	0	0, 0	0	0, 0	0	0, 0	0	0, 0	0	0, 0	0	0, 0
**42**	0	NA**	1.34	0.24, 6.86	0	0, 0	0	0, 0	0	0, 0	0	0, 0	0	0, 0	0	0, 0
**46**	0	NA**	1.36	0.24, 6.93	0	0, 0	0	0, 0	0	0, 0	0	0, 0	0	0, 0	0	0, 0
**7A**	0	NA**	1.34	0.23, 6.84	0	0, 0	0	0, 0	0	0, 0	0	0, 0	0	0, 0	0	0, 0
**7C**	0	NA**	1.42	0.47, 4.39	0	0, 0	0	0, 0	0	0, 0	0	0, 0	0	0, 0	0	0, 0

**All**					167	88, 245	17	10, 26	49	29, 70	149	94, 203	242	162, 320	624	397, 850

carr. = carriage; CrI = 95% credible interval; med. = median; PR = prevalence ratio.

a * Indicates that the prevalence at Time 0 was 0, and hence the observed prevalence ratio could not be estimated for that serotype. In instances where the prevalence at Time 6 was also 0, there is an additional asterisk (**).

b Note that the total cases gained in the last row adds up to more than the number of cases above for each serotype, because the serotypes shown are only those used in the carriage model. The values for total cases gained also included calculations for additional serotypes (not shown) that were responsible for IPD, but were not present in carriage in children under 5 during this time period.

Serotype 12F was responsible for 112 cases gained as a result of increased carriage in children out of the total 394 IPD cases for 12F. In all age groups, 12F was among the highest ranked serotypes among cases gained as a result of increased carriage in children. Serotype 8 was similarly highly ranked for cases gained as a result of increased carriage in children in all age groups except for children <5 years. Serotype 16F was highly ranked among cases gained only among adults 18 years and older.

Adults 65+ and children under five years were responsible for the most IPD cases (Table 1, Fig. 3). In general, observed VT cases decreased over the study period, whereas the additional cases gained (explained and unexplained) increased over the study period.

We had intended to compare results from carriage models with younger and older children to assess whether changes in carriage in different age groups better correlated with changes in IPD in adults; however, the data were too sparse for a formal comparison.

### 3.5 Cases not explained by carriage

The number of additional cases gained over this time period that were not explained by increased carriage in children was also substantial. Over the study period, an estimated 462 (CI: 312, 641) cases were not predicted based on changes in carriage in children. The serotypes with the most unexplained cases gained were 12F, 24F, 22F, 10A, and 15BC (Table 2). Serotype 12F had the most unexplained cases, with an estimated 156 (CI: 50, 268) cases.

**Table 2:** Estimated additional cases gained that are not explained by increased carriage in children. The median and 95% credible intervals for the unexplained excess cases during the full time period and across all ages is shown for each serotype.

**Serotype**	**Median**	**CrI**
12F	156	50, 268
24F	53	25, 80
22F	42	16, 69
10A	30	19, 47
15BC	22	5, 36
6C	19	16, 22
16F	19	0, 55
15A	19	2, 39
33F	18	0, 53
2	14	0, 23
10B	13	3, 25
31	6	1, 20
13	6	1, 10
7B	5	1, 16
23A	5	0, 13
17F	3	0, 10
24B	3	1, 4
11A	3	0, 12
29	3	1, 4
34	2	0, 7
18A	2	0, 3
37	2	0, 2
16	2	1, 2
18B	2	1, 2
10F	1	0, 2
25A	1	0, 2
39	1	1, 2
35F	1	0, 1
35A	1	0, 1
11C	1	0, 1
8	1	0, 28
28A	1	0, 1
25F	1	1, 1
9L	1	0, 1
6D	1	0, 1
22A	1	1, 1
28F	1	1, 1
19B	1	0, 1
27	1	0, 6
23B	0	0, 6
10C	0	0, 0
11B	0	0, 0
12A	0	0, 0
15F	0	0, 0
19C	0	0, 0
20	0	0, 2
21	0	0, 0
24A	0	0, 0
33A	0	0, 0
33B	0	0, 0
33D	0	0, 0
35B	0	0, 0
35C	0	0, 0
38	0	0, 0
42	0	0, 0
46	0	0, 0
7	0	0, 0
7A	0	0, 0
7C	0	0, 0
9A	0	0, 0
9N	0	0, 8

CrI = 95% credible interval.

## 4 Discussion

With several higher-valency PCVs under development, there is a need to understand and predict the patterns of serotype replacement to anticipate future changes. In this study, we created a model that quantifies changes in serotype prevalence post-PCV introduction in Israel using carriage data. We found that the common assumption that NVTs increase by the same proportion overestimates changes in serotype prevalence in Jewish and Bedouin children. Instead, we found that the change in serotype prevalence over time has a positively skewed distribution. Using pre-vaccine prevalence data, we can estimate where on the distribution of post-vaccine changes serotypes will fall. We can then combine observed IPD estimates and model-estimated prevalence ratios to get an estimate of serotype-specific replacement due to increased carriage. We were able to rank serotypes based on cases gained as a result of increased carriage in children. However, there were also additional cases gained over this study period that were not predicted by increased carriage, suggesting the need to explore additional mechanisms.

The estimated number of cases that were not explained by the increased carriage in children illuminated some of the limitations of using carriage alone to predict disease. Changes in carriage prevalence explained some of the variations in IPD in adults, but not all. Many excess IPD cases occurred during this time period that were not predicted by increased carriage. Surprisingly, serotype 8 did not have many unexplained cases. It has recently been seen to be one of the most important serotypes to emerge in other populations, particularly in older age groups.

Changes like serotype-specific epidemics cycles or changes in ascertainment could account for some of this variability. Moreover, carriage patterns in specific age groups (e.g., older children, adults) might better reflect the changes in IPD seen in these other age groups. We had intended to carry out this comparison, but our data were too sparse in subsets of the population. Future work could investigate this difference further.

We found that pre-vaccine prevalence was correlated with where on the distribution of slopes serotypes are located, with serotypes having higher pre-vaccine prevalence associated with larger increases after vaccination. In other words, more prevalent serotypes before introduction of PCV-13 were associated with larger increases over the study period.

Our method of ranking serotypes varied between age groups, but overall the same serotypes had the highest number of cases gained through serotype replacement after vaccination. Unsurprisingly, serotype 8 was among the highest serotypes in this ranking system. Also not surprising were the high rankings of serotypes 12F and 33F, which have been noted to increase in recent years.

Recent work has shown that models that incorporate genomics or clonal groups can forecast which serotypes will be successful after vaccination [19], [20], [33]. Future work could combine the results of this study with new genomics studies, directly incorporating information on the fitness of a serotype (e.g., as calculated by Azarian) in these models, or this type of model structure could be used to benchmark alternative modeling approaches.

A fundamental limitation of using carriage data to forecast changes in IPD is that changes in serotypes that are rarely carried in children cannot be easily tracked. It is not clear if serotypes with these characteristics, like serotypes 1, 5, and 8, might be transmitted directly among older age groups (bypassing children) or if they might be transmitted between symptomatic individuals.

This study had other limitations. Many serotypes are rare, and as a result we did not have estimates for all serotypes for all variables. However, this lack of data is not likely to impact the results greatly because those serotypes are so rare in carriage and disease and would probably not factor into the model. Additionally, the possibility of cross-protection conferred by the vaccine between members of the same serogroups (for example, 6B in PCV7 can prevent IPD caused by 6A) may have impacted carriage and IPD rates.

This analysis was limited to the data in a single country. The dataset contained only seven years of data, and only included carriage data from children. The model structure additionally had some assumptions. We attempted to address these structural assumptions by comparing several models (variations in random and fixed effects, covariance matrices, predictors, and time). When compared to the observed data, the model appeared to fit, suggesting that these assumptions were valid. Future research could investigate these assumptions further, using other populations and additional time periods. Additionally, the IPD data available were comprised of mostly Jewish individuals. As a result, we were unable to extrapolate further for the Bedouin population past fitting the carriage model. Further analyses could explore higher transmission populations.

Though we identified some of the top serotypes using our method of ranking, it is important to note that looking at individual serotypes can be sometimes misleading. Previous work demonstrates that estimating changes for specific serotypes can be subject to a high degree of noise, sometimes leading to inaccurate projections [17]. Future work could incorporate additional predictors of serotype prevalence growth as they become available.

Having a strong understanding of the patterns of serotype replacement could be important as newer higher-valent vaccines are developed and introduced. With higher-valency vaccines, we need to be able to predict both the decline in VT and the increase in NVT serotypes. We can use this model to quantify changes in serotype replacement in other populations using the same analysis. This model may also help to optimize future serotype compositions.

## Data and code availability

All code are available online at <https://github.com/mailephillips/post-pcv-expansion**>.** Due to issues of privacy and data sharing, the data shared on GitHub are not the original data. Those data have been simulated.

## Funding

This work was supported by the National Institutes of Health/National Institute of Allergy and Infectious Diseases [grant numbers R01-AI123208, R01-AI137093] and the Bill and Melinda Gates Foundation (OPP1176267). The funding agency was not involved in the design and conduct of the study; collection, management, analysis, and interpretation of the data; preparation, review, or approval of the manuscript; and decision to submit the manuscript for publication. The corresponding author had full access to all the data in the study and had final responsibility for the decision to submit for publication.

## CRediT authorship contribution statement

**Maile T. Phillips:** Methodology, Formal analysis, Writing - original draft, Writing - review & editing. **Joshua L. Warren:** Methodology, Validation, Writing - review & editing. **Noga Givon-Lavi:** Data curation. **Adrienn Tothpal:** Data curation, Writing - review & editing. **Gili Regev-Yochay:** Data curation, Writing - review & editing. **Ron Dagan:** Data curation, Writing - review & editing. **Daniel M. Weinberger:** Conceptualization, Methodology, Validation, Writing - review & editing, Supervision.

## Declaration of Competing Interest

The authors declare the following financial interests/personal relationships which may be considered as potential competing interests: [DMW has received consulting fees from Pfizer, Merck, GSK, and Affinivax, and is Principal Investigator on a grant from Pfizer to Yale University. RD has received consulting fees from Pfizer, MSD and MeMed; research grants from Pfizer and MSD; speaker fees from Pfizer. GRY has received consulting fees and research funding from Pfizer and research support from GSK. All other co-authors declare no potential conflict of interest.].
