# Supplementary material for: Evaluating post-vaccine expansion patterns of pneumococcal serotypes
Source: Vaccine. 2020 Nov 17;38(49):7756–63. doi: 10.1016/j.vaccine.2020.10.045 (PMC7664987; doi:10.1016/j.vaccine.2020.10.045)
Supplement: Supplementary data 2 [file mmc2.docx]

**S1 Text. Carriage model**

The final carriage model fit took the form of hierarchical multinomial regression with a covariance matrix to model the relationship between pre-vaccine prevalence and the post-vaccine change. The full model, including all priors and hyperpriors, is defined as:

**Main model:**

$${n.carr}_{t}\sim Multinomial\left( {N.swab}_{t}; {prev_{rt},prev}_{1t},{prev}_{2t}, \ldots,{prev}_{it},\ldots,{prev}_{mt} \right)$$

$$\ln\left( \frac{{prev}_{it}}{{prev}_{rt}} \right)=\beta_{0,i}+\beta_{1,i}*time_{1,t}+\beta_{2,i}*time_{2,t}$$

**Priors:**

$$\left[ \begin{aligned} \beta_{0,i} \\ \beta_{2,i} \end{aligned} \right]\sim MVN\left( \left[ \begin{aligned} \beta_{0} \\ \beta_{2} \end{aligned} \right],\left[ \begin{matrix} \sigma_{0}^{2} & \rho\sigma_{0}\sigma_{2} \\ \rho\sigma_{0}\sigma_{2} & \sigma_{2}^{2} \end{matrix} \right] \right)$$

$$\beta_{1,i}\sim N\left( \beta_{1}, \sigma_{\beta_{1}}^{2} \right)$$

$$\rho\sim U(-1,1)$$

**Hyperpriors:**

$$\beta_{0}\sim N(0, 100,000)$$

$$\beta_{1}\sim N(0, 100,000)$$

$$\mu_{0}\sim N(0, 100,000)$$

$$\sigma_{\beta_{0}}\sim U(0, 100)$$

$$\sigma_{\beta_{1}}\sim U(0, 100)$$

$$\sigma_{\mu_{0}}\sim U(0, 100)$$

*n.carr_t_* = pneumococcal carriage count at time period *t*

*N.swab_t_* = total # swabs at time period *t*

*prev_it_*  = pneumococcal prevalence for non-vaccine serotype *i* at time

period *t* (m=54 non-vaccine serotypes)

$prev_{rt}$ = pneumococcal prevalence from the “reference” group at time

period *t* (“reference” swab is defined as negative for any pneumococcus or positive for vaccine-serotypes)

*i*  = 1, 2, …, m

*t* = 0, 1, …, 6 (year)

*time_1_* = 0, 1, 1, 1, 1, 1, 1

*time_2_* = 0, 0, 0, 1, 1, 1, 1

**Imputation**

Many serotypes are rare, and as a result are not commonly observed. We did not have estimates for all serotypes, but did not want to restrict our analysis to only serotypes for which we had observed data for all variables. Instead, we restricted our carriage dataset to include all serotypes observed in either carriage or covariate datasets at any given time point. If a serotype was observed in the covariate dataset but not the carriage dataset, the value for that serotype was set to zero carriage counts for all time periods. This approach resulted in a final dataset of 54 non-vaccine serotypes in the carriage model. To estimate cases gained, we used any serotypes that appeared either in the carriage model or in the IPD dataset (61 non-vaccine serotypes).

For predictors in the carriage model, we assumed the data were missing completely at random. In other words, we assumed that the missing values were similar to the non-missing values. To impute the missing values in the dataset, in the Bayesian model we fit a weakly informative normal distribution around the observed values, and then imputed the missing values using the same normal distribution estimated for the observed values.

**Comparison of model structures**

We compared many variations of the carriage model before choosing our final model. We examined different assumptions about model structure and covariates. For model structure, we fit models with different assumptions about time, allowing time to be linear or categorical. For categorical time, after examining patterns in the raw data, we allowed each time point to be modeled independently (seven categories), and we allowed time to have separate pre- and post-vaccination periods with time for prevalence to level out (both with separate and overlapping second-category time periods).

We also fit models with all variations of fixed and random effects for the intercepts and slopes in the regression part of the model ($\ln\left( \frac{{prev}_{it}}{{prev}_{rt}} \right)=\beta_{0,i}+\beta_{1,i}*time_{1,t}+\beta_{2,i}*time_{2,t}$). When it was determined that all parameters fit best with serotype-specific effects, we also compared a model with and without a covariance matrix for the pre- and post-vaccination parameters to model dependence or independence of the prevalence during the different time periods.

We also compared models with variables as predictors of different slopes or intercepts ($\beta_{0,i}$, $\beta_{1,i}$, or $\beta_{2,i}$). We additionally fit all of the previously mentioned model variations with all combinations of predictors (including no predictors).

The best model formulation was chosen using the optimal deviance information criterion (DIC).

**Model diagnostics**

Using Markov chain Monte Carlo sampling techniques, we obtained 150,000 posterior samples from the joint posterior distribution following a burn-in period of 10,000 iterations. The Gelman-Rubin (1) and Geweke (2) diagnostics were used to investigate convergence of individual parameters and effective sample sizes were calculated to ensure we collected enough posterior samples post-convergence to make accurate inference. We observed that 97% of the Geweke diagnostic Z-scores fell between -1.96 and 1.96, and found adequate effective sample sizes (greater than 1,000) across all models and parameters.

**Figure S1. Observed and expected prevalence plot, by year (Jewish children).** *Each panel shows the observed (black points) and model-fitted estimates of prevalence (different-colored dots and bars represent point estimates and 95% credible intervals), by year of the study period.*

**Figure S2. Observed and expected prevalence plot, by year (Bedouin children).** *Each panel shows the observed (black points) and model-fitted estimates of prevalence (different-colored dots and bars represent point estimates and 95% credible intervals), by year of the study period.*

**Figure S3. Observed versus fitted prevalence ratios (Jewish children).** *The plot shows the observed (black points) and model-fitted prevalence ratios (different-colored dots and bars represent point estimates and 95% credible intervals). The size of the different-colored dots indicates the number of isolates over the study period from the raw data (smaller dots = less isolates, larger dots = more isolates). Ratios shown are for the first to last years of the study period* $\frac{prevalence_{serotype, year6}}{revalence_{serotype, year0}}$ *. In instances where the observed prevalence in both Year 0 and Year 6 were 0, the observed prevalence ratio is indicated to be one. In cases where the observed prevalence in Year 0 was 0 and but the observed prevalence in Year 6 was nonzero, no observed point is shown (serotypes 20, 27, 29, and 7B).*

**Fig S4. Observed versus fitted prevalence ratios (Bedouin children).** *The plot shows the observed (black points) and model-fitted prevalence ratios (different-colored dots and bars represent point estimates and 95% credible intervals). The size of the different-colored dots indicates the number of isolates over the study period from the raw data (smaller dots = less isolates, larger dots = more isolates). Ratios shown are for the first to last years of the study period* $\frac{prevalence_{serotype, year6}}{revalence_{serotype, year0}}$ *.*

**Figure S5. Estimates for log relative risk ratios for pre-PCV period.** *Each serotype-specific log relative risk ratio with its 95% credible interval is shown for the pre-PCV period (*$\beta_{0,i}$*), estimated from the model. The serotypes are shown from highest to lowest log relative risk ratios, separately for Jewish (A) and Bedouin (B) children.*

**A)**

**B)**

**Figure S6. Estimates for log relative risk ratios for early post-PCV period.** *Each serotype-specific log relative risk ratio with its 95% credible interval is shown for the early post-PCV period (*$\beta_{1,i}$*), estimated from the model. The serotypes are shown from highest to lowest log relative risk ratios, separately for Jewish (A) and Bedouin (B) children.*

**A)**

**B)**

**Figure S7. Estimates for log relative risk ratios for late post-PCV period.** *Each serotype-specific log relative risk ratio with its 95% credible interval is shown for the late post-PCV period (*$\beta_{2,i}$*), estimated from the model. The serotypes are shown from highest to lowest log relative risk ratios, separately for Jewish (A) and Bedouin (B) children.*

**A)**

**B)**

**Figure S8. Estimated additional cases gained as a result of increased carriage in children, by serotype.** *Each panel shows an individual serotype over the study period for all ages. Each full bar represents the total number of observed IPD cases for that stratum. The different colors denote the fraction of total cases attributable to different groups of serotypes: expected non-vaccine-targeted serotype cases had PCV13 not been introduced (black wide diagonal hatches), estimated additional cases gained that are not explained by this model (black horizontal hatches), and the estimated additional cases gained as a result of increased carriage in children* *(light grey narrow diagonal hatches).*


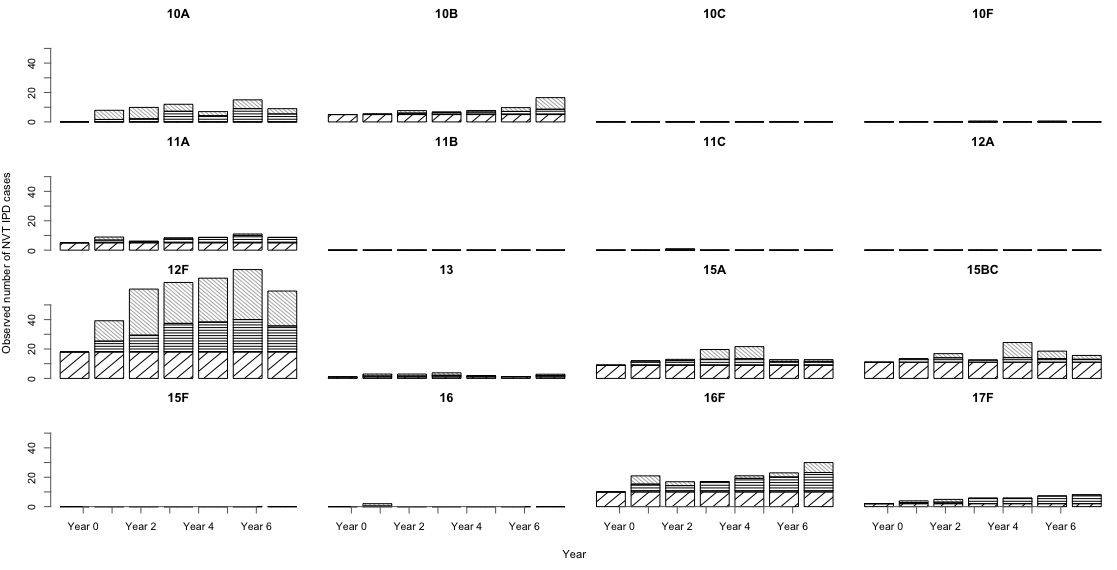


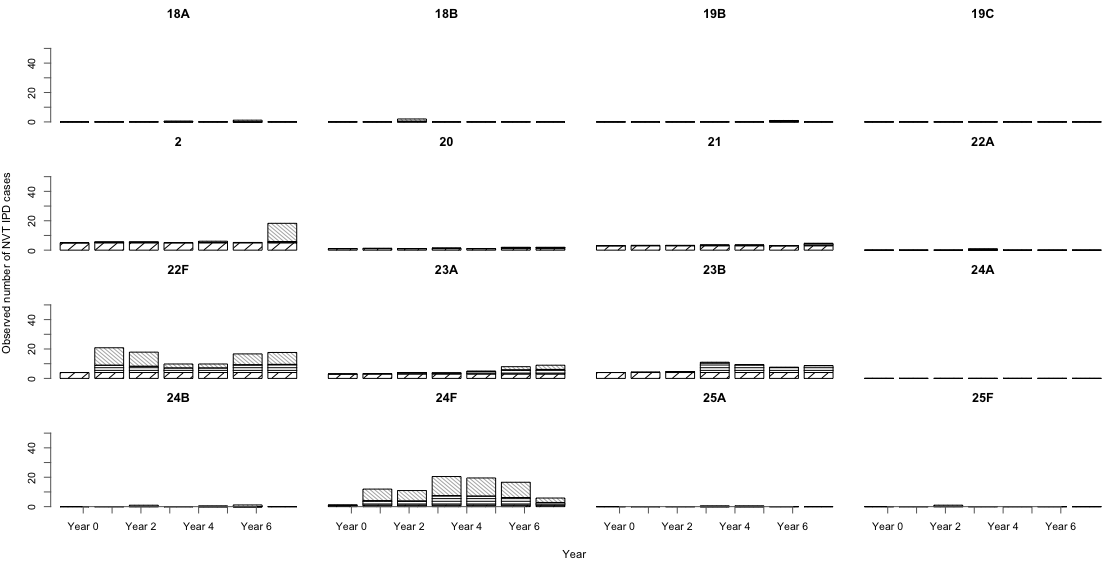


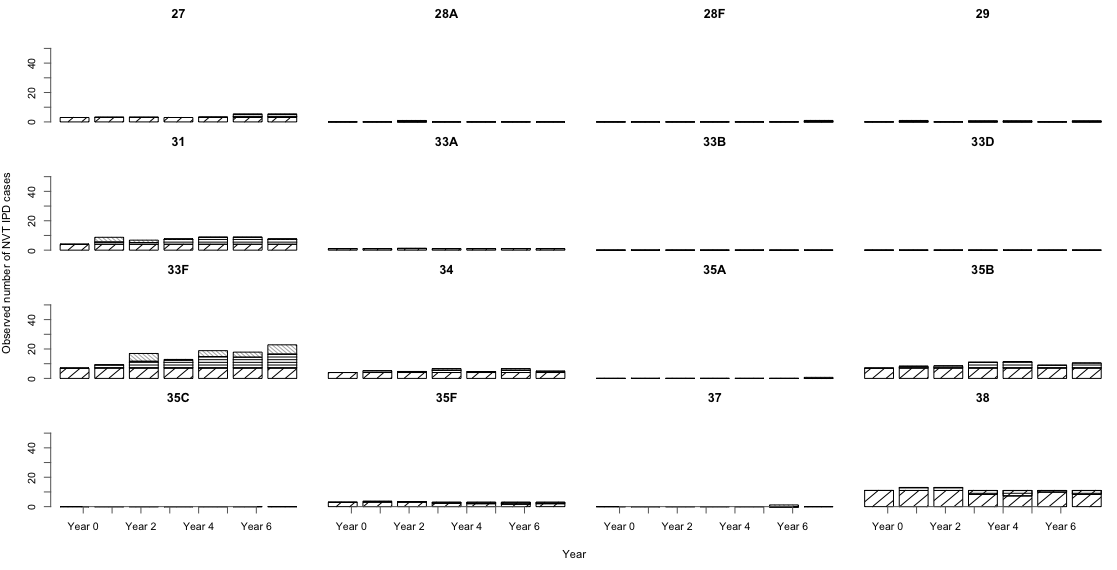


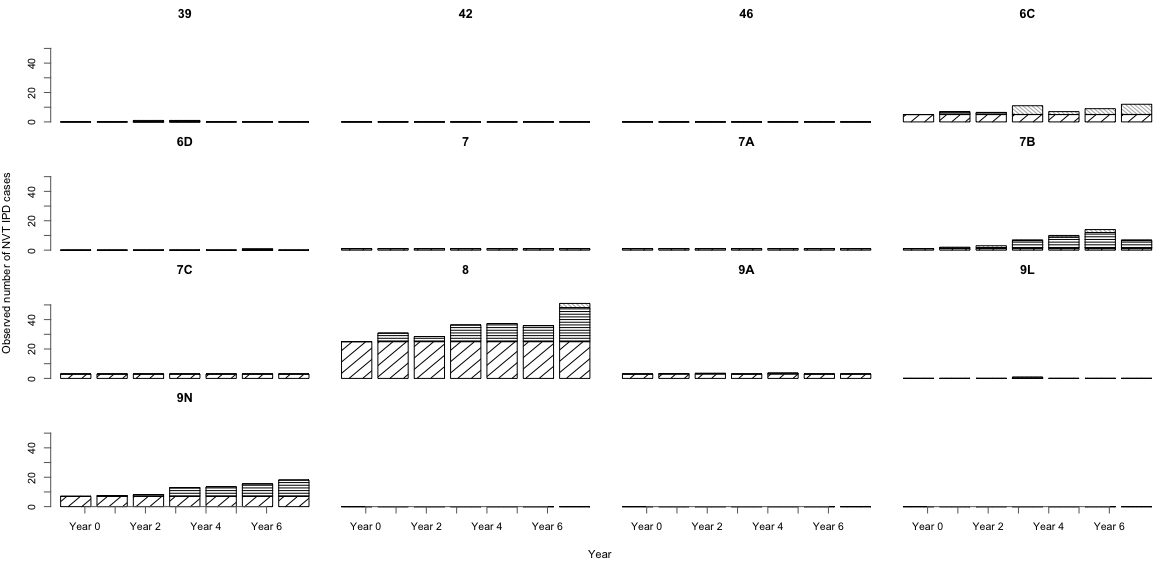


**Table S1. Penalized deviance for all model combinations fit.** *The deviance information criterion (DIC) used to determine the best fit model is shown for all combinations of models for both Jewish and Bedouin datasets. An “X” indicates whether that particular feature was used in building the specified model.*

|  | **Covariance matrix** | **Predictors** | | | **DIC** | |
| --- | --- | --- | --- | --- | --- | --- |
| **Model** |  | *CFR* | *Density* | *TCR* | *Jewish* | *Bedouin* |
| **1** | X |  |  |  | 994 | 1324 |
| **2** |  |  |  |  | 1000 | 1322 |
| **3** |  | X |  |  | 1058 | 1388 |
| **4** |  |  | X |  | 1058 | 1386 |
| **5** |  |  |  | X | 1065 | 1396 |
| **6** |  | X | X |  | 1123 | 1452 |
| **7** |  | X |  | X | 1132 | 1462 |
| **8** |  |  | X | X | 1130 | 1460 |
| **9** |  | X | X | X | 1196 | 1526 |

**Table S2. Serotype-specific parameters estimated from model: Jewish children under 5 in Israel, November 2009-July 2016.**

*Estimates for the serotype-specific pre-PCV relative risk ratios (RRRs) (*$e^{\beta_{0,i}}$*), early post-PCV RRRs (*$e^{\beta_{1,i}}$*), and late post-PCV RRRs (*$e^{\beta_{2,i}}$*) are shown for the Jewish carriage model, with their 95% credible intervals.*

|  | **Jewish carriage model** | | | | | |
| --- | --- | --- | --- | --- | --- | --- |
|  | $e^{\beta_{0,i}}$ | | $e^{\beta_{1,i}}$ | | $e^{\beta_{2,i}}$ | |
| **Serotype** | *med.* | *CrI* | *med.* | *CrI* | *med.* | *CrI* |
| **10A** | 0.01 | 0.00, 0.01 | 1.38 | 0.91, 2.19 | 2.24 | 1.28, 4.25 |
| **10B** | 0.02 | 0.01, 0.03 | 1.29 | 0.76, 1.84 | 1.24 | 0.77, 2.00 |
| **10C** | 0.00 | 0.00, 0.00 | 1.37 | 0.84, 2.18 | 1.17 | 0.21, 5.66 |
| **10F** | 0.00 | 0.00, 0.00 | 1.37 | 0.84, 2.20 | 1.16 | 0.21, 5.77 |
| **11A** | 0.02 | 0.01, 0.03 | 1.38 | 0.93, 2.08 | 1.64 | 1.06, 2.59 |
| **11B** | 0.00 | 0.00, 0.00 | 1.37 | 0.84, 2.19 | 1.17 | 0.22, 5.66 |
| **11C** | 0.00 | 0.00, 0.00 | 1.37 | 0.83, 2.19 | 1.17 | 0.21, 5.73 |
| **12A** | 0.00 | 0.00, 0.00 | 1.38 | 0.86, 2.24 | 1.38 | 0.36, 5.72 |
| **12F** | 0.01 | 0.00, 0.01 | 1.35 | 0.84, 2.04 | 1.31 | 0.70, 2.48 |
| **13** | 0.00 | 0.00, 0.01 | 1.38 | 0.89, 2.25 | 1.32 | 0.56, 3.19 |
| **15A** | 0.02 | 0.02, 0.04 | 1.42 | 1.00, 2.18 | 1.06 | 0.72, 1.58 |
| **15BC** | 0.05 | 0.03, 0.07 | 1.33 | 0.93, 1.85 | 1.03 | 0.76, 1.41 |
| **15F** | 0.00 | 0.00, 0.00 | 1.37 | 0.84, 2.19 | 1.17 | 0.21, 5.81 |
| **16F** | 0.01 | 0.01, 0.02 | 1.44 | 1.00, 2.42 | 1.49 | 0.95, 2.40 |
| **17F** | 0.00 | 0.00, 0.01 | 1.38 | 0.89, 2.24 | 3.69 | 1.67, 10.56 |
| **18A** | 0.00 | 0.00, 0.00 | 1.37 | 0.83, 2.19 | 1.17 | 0.21, 5.68 |
| **19B** | 0.00 | 0.00, 0.01 | 1.39 | 0.89, 2.26 | 1.39 | 0.61, 3.38 |
| **19C** | 0.00 | 0.00, 0.00 | 1.37 | 0.83, 2.18 | 1.17 | 0.22, 5.72 |
| **2** | 0.00 | 0.00, 0.00 | 1.35 | 0.79, 2.09 | 1.17 | 0.32, 3.95 |
| **20** | 0.00 | 0.00, 0.00 | 1.38 | 0.88, 2.28 | 1.71 | 0.56, 6.16 |
| **21** | 0.01 | 0.00, 0.02 | 1.38 | 0.92, 2.17 | 1.90 | 1.12, 3.37 |
| **22F** | 0.01 | 0.00, 0.01 | 1.42 | 0.96, 2.39 | 1.25 | 0.68, 2.35 |
| **23A** | 0.02 | 0.01, 0.03 | 1.41 | 0.98, 2.24 | 1.30 | 0.86, 1.99 |
| **23B** | 0.02 | 0.01, 0.02 | 1.40 | 0.95, 2.16 | 2.08 | 1.36, 3.27 |
| **24A** | 0.00 | 0.00, 0.00 | 1.38 | 0.87, 2.26 | 1.01 | 0.23, 3.72 |
| **24B** | 0.00 | 0.00, 0.00 | 1.37 | 0.84, 2.19 | 1.17 | 0.21, 5.78 |
| **24F** | 0.01 | 0.00, 0.01 | 1.43 | 0.98, 2.53 | 1.24 | 0.65, 2.44 |
| **25A** | 0.00 | 0.00, 0.00 | 1.37 | 0.84, 2.19 | 1.17 | 0.22, 5.62 |
| **27** | 0.00 | 0.00, 0.00 | 1.38 | 0.87, 2.27 | 1.56 | 0.47, 5.88 |
| **28A** | 0.00 | 0.00, 0.00 | 1.37 | 0.83, 2.19 | 1.17 | 0.22, 5.70 |
| **29** | 0.00 | 0.00, 0.00 | 1.38 | 0.85, 2.24 | 1.38 | 0.36, 5.62 |
| **31** | 0.01 | 0.00, 0.01 | 1.34 | 0.80, 1.99 | 1.97 | 1.06, 3.95 |
| **33A** | 0.00 | 0.00, 0.00 | 1.39 | 0.90, 2.33 | 1.10 | 0.35, 3.24 |
| **33B** | 0.00 | 0.00, 0.00 | 1.37 | 0.84, 2.19 | 1.17 | 0.21, 5.76 |
| **33D** | 0.00 | 0.00, 0.00 | 1.37 | 0.84, 2.20 | 1.17 | 0.21, 5.67 |
| **33F** | 0.01 | 0.00, 0.01 | 1.46 | 1.02, 2.82 | 1.41 | 0.76, 2.74 |
| **34** | 0.01 | 0.00, 0.01 | 1.41 | 0.96, 2.36 | 1.13 | 0.60, 2.15 |
| **35A** | 0.00 | 0.00, 0.00 | 1.39 | 0.89, 2.30 | 0.99 | 0.28, 3.02 |
| **35B** | 0.01 | 0.01, 0.02 | 1.39 | 0.94, 2.13 | 1.73 | 1.11, 2.77 |
| **35C** | 0.00 | 0.00, 0.00 | 1.37 | 0.84, 2.20 | 1.17 | 0.22, 5.79 |
| **35F** | 0.00 | 0.00, 0.01 | 1.39 | 0.90, 2.21 | 0.71 | 0.31, 1.52 |
| **37** | 0.00 | 0.00, 0.00 | 1.35 | 0.79, 2.07 | 1.01 | 0.23, 3.71 |
| **38** | 0.01 | 0.01, 0.02 | 1.35 | 0.86, 2.03 | 0.69 | 0.35, 1.29 |
| **42** | 0.00 | 0.00, 0.00 | 1.37 | 0.84, 2.18 | 1.17 | 0.21, 5.74 |
| **46** | 0.00 | 0.00, 0.00 | 1.37 | 0.83, 2.17 | 1.18 | 0.22, 5.79 |
| **6C** | 0.02 | 0.01, 0.03 | 1.41 | 0.98, 2.22 | 0.49 | 0.27, 0.85 |
| **6D** | 0.00 | 0.00, 0.00 | 1.38 | 0.88, 2.27 | 1.16 | 0.32, 3.97 |
| **7A** | 0.00 | 0.00, 0.00 | 1.37 | 0.84, 2.20 | 1.16 | 0.21, 5.73 |
| **7B** | 0.00 | 0.00, 0.01 | 1.42 | 0.95, 2.50 | 4.10 | 1.85, 11.78 |
| **7C** | 0.00 | 0.00, 0.00 | 1.40 | 0.91, 2.35 | 1.20 | 0.41, 3.48 |
| **8** | 0.00 | 0.00, 0.00 | 1.40 | 0.91, 2.34 | 1.66 | 0.61, 5.11 |
| **9A** | 0.00 | 0.00, 0.00 | 1.37 | 0.84, 2.19 | 1.17 | 0.22, 5.82 |
| **9L** | 0.00 | 0.00, 0.00 | 1.37 | 0.84, 2.19 | 1.17 | 0.21, 5.79 |
| **9N** | 0.00 | 0.00, 0.01 | 1.39 | 0.91, 2.29 | 2.61 | 1.23, 6.42 |
| **NEG+VTs** | REF | | REF | | REF | |
| CrI=95% credible interval; med.=median estimate; NEG=negative swab result; REF=reference; VTs=vaccine serotypes | | | | | | |

**Table S3. Serotype-specific parameters estimated from model: Bedouin children under 5 in Israel, November 2009-July 2016.**

*Estimates for the serotype-specific pre-PCV relative risk ratios (RRRs) (*$e^{\beta_{0,i}}$*), early post-PCV RRRs (*$e^{\beta_{1,i}}$*), and late post-PCV RRRs (*$e^{\beta_{2,i}}$*) are shown for the Bedouin carriage model, with their 95% credible intervals.*

|  | **Bedouin carriage model** | | | | | |
| --- | --- | --- | --- | --- | --- | --- |
|  | $e^{\beta_{0,i}}$ | | $e^{\beta_{1,i}}$ | | $e^{\beta_{2,i}}$ | |
| **Serotype** | *med.* | *CrI* | *med.* | *CrI* | *med.* | *CrI* |
| **10A** | 0.01 | 0.00, 0.01 | 1.59 | 0.96, 2.56 | 0.82 | 0.48, 1.36 |
| **10B** | 0.01 | 0.00, 0.01 | 1.73 | 1.18, 4.03 | 1.35 | 0.80, 2.42 |
| **10C** | 0.00 | 0.00, 0.00 | 1.58 | 0.80, 2.66 | 1.06 | 0.37, 2.88 |
| **10F** | 0.00 | 0.00, 0.01 | 1.67 | 1.07, 3.45 | 1.70 | 0.89, 3.69 |
| **11A** | 0.02 | 0.01, 0.03 | 1.57 | 1.01, 2.33 | 0.99 | 0.70, 1.43 |
| **11B** | 0.00 | 0.00, 0.00 | 1.58 | 0.79, 2.68 | 1.28 | 0.48, 3.71 |
| **11C** | 0.00 | 0.00, 0.00 | 1.57 | 0.75, 2.62 | 1.20 | 0.42, 3.59 |
| **12A** | 0.00 | 0.00, 0.00 | 1.56 | 0.69, 2.52 | 1.13 | 0.35, 3.59 |
| **12F** | 0.00 | 0.00, 0.01 | 1.69 | 1.12, 3.59 | 0.81 | 0.41, 1.52 |
| **13** | 0.01 | 0.00, 0.01 | 1.61 | 1.00, 2.64 | 1.29 | 0.81, 2.12 |
| **15A** | 0.02 | 0.01, 0.03 | 1.54 | 0.94, 2.24 | 0.96 | 0.67, 1.41 |
| **15BC** | 0.04 | 0.03, 0.06 | 1.62 | 1.15, 2.33 | 0.91 | 0.70, 1.18 |
| **15F** | 0.00 | 0.00, 0.00 | 1.57 | 0.75, 2.61 | 1.20 | 0.42, 3.62 |
| **16F** | 0.03 | 0.02, 0.04 | 1.71 | 1.22, 2.86 | 1.12 | 0.83, 1.52 |
| **17F** | 0.04 | 0.02, 0.05 | 1.60 | 1.10, 2.33 | 0.84 | 0.63, 1.12 |
| **18A** | 0.00 | 0.00, 0.00 | 1.54 | 0.75, 2.43 | 1.01 | 0.48, 2.14 |
| **19B** | 0.01 | 0.00, 0.02 | 1.56 | 0.88, 2.42 | 0.84 | 0.50, 1.38 |
| **19C** | 0.00 | 0.00, 0.00 | 1.57 | 0.75, 2.62 | 1.20 | 0.42, 3.60 |
| **2** | 0.00 | 0.00, 0.00 | 1.59 | 0.83, 2.76 | 1.35 | 0.54, 3.84 |
| **20** | 0.00 | 0.00, 0.00 | 1.64 | 0.98, 3.08 | 1.10 | 0.51, 2.40 |
| **21** | 0.01 | 0.01, 0.02 | 1.71 | 1.19, 3.28 | 1.03 | 0.69, 1.55 |
| **22F** | 0.00 | 0.00, 0.01 | 1.72 | 1.16, 3.92 | 0.98 | 0.55, 1.78 |
| **23A** | 0.01 | 0.01, 0.02 | 1.62 | 1.04, 2.69 | 0.98 | 0.64, 1.52 |
| **23B** | 0.02 | 0.01, 0.03 | 1.78 | 1.27, 3.63 | 1.05 | 0.75, 1.48 |
| **24A** | 0.00 | 0.00, 0.00 | 1.57 | 0.74, 2.61 | 0.98 | 0.32, 2.77 |
| **24B** | 0.00 | 0.00, 0.00 | 1.57 | 0.75, 2.58 | 0.98 | 0.32, 2.77 |
| **24F** | 0.01 | 0.00, 0.01 | 1.63 | 1.02, 2.95 | 1.03 | 0.59, 1.84 |
| **25A** | 0.00 | 0.00, 0.00 | 1.57 | 0.75, 2.60 | 1.20 | 0.42, 3.59 |
| **27** | 0.00 | 0.00, 0.00 | 1.57 | 0.79, 2.56 | 1.02 | 0.44, 2.37 |
| **28A** | 0.00 | 0.00, 0.00 | 1.55 | 0.73, 2.50 | 1.07 | 0.43, 2.65 |
| **29** | 0.00 | 0.00, 0.00 | 1.59 | 0.82, 2.71 | 1.36 | 0.54, 3.85 |
| **31** | 0.00 | 0.00, 0.01 | 1.70 | 1.14, 3.74 | 1.17 | 0.64, 2.21 |
| **33A** | 0.00 | 0.00, 0.00 | 1.54 | 0.66, 2.43 | 1.13 | 0.43, 2.96 |
| **33B** | 0.00 | 0.00, 0.00 | 1.58 | 0.80, 2.68 | 1.28 | 0.48, 3.74 |
| **33D** | 0.00 | 0.00, 0.00 | 1.57 | 0.74, 2.62 | 1.20 | 0.42, 3.61 |
| **33F** | 0.01 | 0.01, 0.02 | 1.63 | 1.04, 2.71 | 0.73 | 0.44, 1.18 |
| **34** | 0.01 | 0.01, 0.02 | 1.58 | 0.98, 2.41 | 0.90 | 0.59, 1.37 |
| **35A** | 0.00 | 0.00, 0.00 | 1.57 | 0.75, 2.60 | 0.98 | 0.32, 2.76 |
| **35B** | 0.04 | 0.02, 0.05 | 1.51 | 0.96, 2.09 | 0.58 | 0.42, 0.81 |
| **35C** | 0.00 | 0.00, 0.00 | 1.61 | 0.90, 2.90 | 1.33 | 0.58, 3.44 |
| **35F** | 0.00 | 0.00, 0.00 | 1.63 | 0.96, 3.07 | 1.60 | 0.75, 4.12 |
| **37** | 0.00 | 0.00, 0.00 | 1.56 | 0.69, 2.54 | 1.13 | 0.34, 3.63 |
| **38** | 0.00 | 0.00, 0.01 | 1.56 | 0.88, 2.50 | 1.09 | 0.61, 1.97 |
| **42** | 0.00 | 0.00, 0.00 | 1.59 | 0.82, 2.75 | 0.93 | 0.34, 2.39 |
| **46** | 0.00 | 0.00, 0.00 | 1.50 | 0.52, 2.24 | 0.78 | 0.26, 1.90 |
| **6C** | 0.01 | 0.00, 0.01 | 1.65 | 1.06, 3.01 | 1.16 | 0.68, 2.03 |
| **6D** | 0.00 | 0.00, 0.00 | 1.50 | 0.55, 2.27 | 0.84 | 0.30, 2.04 |
| **7A** | 0.00 | 0.00, 0.00 | 1.57 | 0.75, 2.60 | 1.20 | 0.42, 3.57 |
| **7B** | 0.00 | 0.00, 0.00 | 1.66 | 1.03, 3.45 | 2.44 | 1.20, 6.32 |
| **7C** | 0.00 | 0.00, 0.00 | 1.54 | 0.69, 2.45 | 1.00 | 0.39, 2.49 |
| **8** | 0.00 | 0.00, 0.00 | 1.60 | 0.89, 2.74 | 0.93 | 0.44, 1.93 |
| **9A** | 0.00 | 0.00, 0.00 | 1.54 | 0.67, 2.44 | 0.78 | 0.26, 1.94 |
| **9L** | 0.00 | 0.00, 0.00 | 1.50 | 0.55, 2.26 | 0.71 | 0.24, 1.69 |
| **9N** | 0.00 | 0.00, 0.01 | 1.69 | 1.12, 3.78 | 3.60 | 1.95, 7.38 |
| **NEG+VTs** | REF | | REF | | REF | |
| CrI=95% credible interval; med.=median estimate; NEG=negative swab result; REF=reference; VTs=vaccine serotypes | | | | | | |

1. Gelman A, Rubin D. Inference from iterative simulation using multiple sequences. *Statistical Science* 1992;7:457-72.

2. Geweke J. *Evaluating the accuracy of sampling-based approaches to calculating posterior moments*. Oxford, UK: Clarendon Press; 1992.
